# Relationship between serum bicarbonate levels and the risk of death within 30 days in ICU patients with acute ischemic stroke

**DOI:** 10.3389/fneur.2023.1125359

**Published:** 2023-05-24

**Authors:** Xia Huang, Yuanyuan Zhang

**Affiliations:** ^1^Department of Neurology, Ninghai First Hospital, Ningbo, Zhejiang, China; ^2^Emergency Medicine Department, Affiliated Hospital of Yangzhou University (Yangzhou First People's Hospital), Yangzhou, Jiangsu, China

**Keywords:** bicarbonate T0, Δbicarbonate, mortality, acute ischemic stroke, MIMIC

## Abstract

**Aim:**

To explore the relationship between baseline bicarbonate levels and their changes with 30-day mortality in patients with acute ischemic stroke who were admitted to the intensive care unit (ICU).

**Methods:**

This cohort study collected the data of 4,048 participants from the Medical Information Mart for Intensive Care (MIMIC)-III and MIMIC-IV databases. Univariate and multivariable Cox proportional risk models were utilized to explore the relationship between bicarbonate T0 and Δbicarbonate with 30-day mortality in patients with acute ischemic stroke. The Kaplan–Meier curves were plotted to measure the 30-day survival probability of patients with acute ischemic stroke.

**Results:**

The median follow-up time was 30 days. At the end of the follow-up, 3,172 patients survived. Bicarbonate T0 ≤ 21 mEq/L [hazard ratio (HR) = 1.24, a 95% confidence interval (CI): 1.02–1.50] or 21 mEq/L < bicarbonate T0 ≤ 23 mEq/L (HR = 1.29, 95%CI: 1.05–1.58) were associated with an increased risk of 30-day mortality in patients with acute ischemic stroke compared with bicarbonate T0 > 26 mEq/L. −2 mEq/L < Δbicarbonate ≤ 0 mEq/L (HR = 1.40, 95%CI: 1.14–1.71), 0 mEq/L < Δbicarbonate ≤ 2 mEq/L (HR = 1.44, 95%CI: 1.17–1.76), and Δbicarbonate >2 mEq/L (HR = 1.40, 95%CI: 1.15–1.71) were correlated with an elevated risk of 30-day mortality in acute ischemic stroke patients. The 30-day survival probability of acute ischemic stroke patients with 21 mEq/L < bicarbonate T0 ≤ 23 mEq/L, 23 mEq/L < bicarbonate T0 ≤ 26 mEq/L, or bicarbonate T0 >26 mEq/L was higher than that of patients with bicarbonate T0 ≤ 21 mEq/L. The 30-day survival probability was greater for patients in the Δbicarbonate ≤ -2 mEq/L group than for those in the Δbicarbonate >2 mEq/L group.

**Conclusion:**

Low baseline bicarbonate levels and decreased bicarbonate levels during the ICU stay were associated with a high risk of 30-day mortality in acute ischemic stroke patients. Special interventions should be offered to those with low baseline and decreased bicarbonate levels during their ICU stay.

## Introduction

Stroke remains one of the leading causes of death and a major cause of disability worldwide (1). Ischemic stroke accounts for 87% of all stroke cases. Acute ischemic stroke is considered a medical emergency due to the decreased blood flow to the brain and is characterized by sudden-onset numbness or weakness in the arm or the leg, facial drooping, difficulty speaking or understanding speech, confusion, trouble with balance or coordination, and the loss of vision (2). Although treatments, including intravenous thrombolysis (IVT) using tissue plasminogen activator (tPA), have improved the functional outcomes of patients with acute ischemic stroke, the prognosis of these patients remains poor (3). Stroke has caused ~5.8 million deaths (4) and is a tremendous financial burden (5). Identifying more reliable biomarkers for predicting the prognosis of patients with acute ischemic stroke could improve patient management and treatment.

In a previous study, endothelial dysfunction was reported to be one of the pathological mechanisms of ischemic stroke (6). Evidence shows that lower serum bicarbonate levels may be associated with endothelial dysfunction, and bicarbonate therapy can improve endothelial function in patients with chronic kidney disease (7, 8). However, researchers have found that peripheral blood electrolyte changes occurred after stroke and that alterations in cerebrovascular acid-base balance directly affected cerebral blood flow (9). Bicarbonate measurements are well-acknowledged as a clinically useful biomarker for assessing the acid-base status to diagnose various disease conditions (10, 11). A previous study indicated that, although there was no statistical significance between the serum bicarbonate levels and the mortality of stroke patients, the serum bicarbonate levels were also considered an important factor that correlated with the prognosis of stroke patients and were included in the prediction model for predicting 30-, 180-, and 360-day survival of stroke patients (12). Other studies have revealed that higher serum bicarbonate levels in patients with hypertension are associated with a higher risk of cardiovascular disease (13, 14). The role of bicarbonate levels in patients with cardiovascular diseases was conflicting. Thus, clarifying the association between serum bicarbonate levels and the risk of death in patients with acute ischemic stroke would be highly valuable. In addition, in severe cases, serum bicarbonate levels can fluctuate with changes in the patient's condition as a result of the treatments received (15). Evaluating the influence of the change in bicarbonate levels on the prognosis of patients with ischemic stroke may be valuable.

In this study, the associations between serum bicarbonate levels and their changes with 30-day mortality in patients with acute ischemic stroke were measured based on the data from the Medical Information Mart for Intensive Care (MIMIC)-III and MIMIC-IV databases. Subgroup analyses were stratified by age, the Charlson comorbidity index (CCI), thrombolysis, antiplatelet agents, and anticoagulation agents.

## Methods

### Study design and population

In this cohort study, 4,674 participants with acute ischemic stroke were identified from the MIMIC-III and MIMIC-IV databases. MIMIC-III is a publicly available single-center critical care database that was approved by the Institutional Review Boards of Beth Israel Deaconess Medical Center (BIDMC, Boston, MA, USA) and the Massachusetts Institute of Technology (Cambridge, MA, USA), and it contains information on 46,520 patients who were admitted to the ICUs of BIDMC in Boston from 2001 to 2012 (16). The information included demographics, vital signs, laboratory tests, fluid balance, and vital status; documents of the International Classification of Diseases and Ninth Revision (ICD-9) codes; records of hourly physiologic data from bedside monitors validated by the ICU nurses; and written evaluations of radiologic films by specialists covering the corresponding time period (17). MIMIC-IV is an updated version of MIMIC-III that features improvements, including a simplified structure, new data elements, and improved usability of previous data elements (18). The project was approved by the Institutional Review Boards of Beth Israel Deaconess Medical Center (Boston, MA) and the Massachusetts Institute of Technology (Cambridge, MA). The use of the data provided by clinicians, data scientists, and information technology personnel, as well as unidentified health information from patients, did not require individual patient consent due to the anonymization of the health information. The diagnosis of acute ischemic stroke was based on ICD-9 (43,301, 43,311, 43,321, 43,331, 43,381, 43,391, 43,401, 43,411, or 43,491) or ICD-10 code (I63). In our study, we excluded individuals under the age of 18 years, those who did not have bicarbonate levels measured at 24-h intervals, and those who died within 2 days of being admitted to the ICU. Finally, 4,048 participants were included, with 3,172 subjects surviving at 30 days and 876 dying within 30 days.

### Potential confounders

Potential confounders analyzed in this study including demographic characteristics, such as age (years), gender (female or male), and ethnicity (Black, White, others or unknown), and clinical data such as ventilation (yes or no), vasopressor (yes or no), coronary artery bypass grafting (CABG) (yes or no), coronary artery disease (yes or no), congestive heart failure (yes or no), peripheral vascular disease (yes or no), hyperlipidemia (yes or no), hypertension (yes or no), chronic kidney disease (yes or no), atrial fibrillation (yes or no), malignant cancer (yes or no), diabetes (yes or no), systolic (mmHg), diastolic (mmHg), respiratory rate (beat/min), heart rate (beat/min), temperature (°C), oxygen saturation (SPO_2_, %), white blood cell (WBC, K/uL), platelet (K/uL), hemoglobin (g/dL), red cell distribution width (RDW, %), blood urea nitrogen (BUN, mg/dL), creatinine (mg/dL), glucose (mg/dL), CCI, simplified acute physiology score II (SAPSII), sequential organ failure assessment (SOFA), quick SOFA (qSOFA), systemic inflammatory response syndrome (SIRS), thrombolysis (yes or no), antiplatelet agents (yes or no), anticoagulation agents, mechanical bolt removal (yes or no), and hemorrhagic transformation (yes or no). The laboratory data were collected within 24 h after admission to the ICU, and drug use was defined as receiving respective drugs at any point during admission.

### Main variables and outcome variables

Bicarbonate T0 (mEq/L), bicarbonate T1 (mEq/L), and Δbicarbonate were the main variables. Bicarbonate T0 was the first measurement of bicarbonate from 0 to 24 h after admission to the ICU, and bicarbonate T1 was the first measurement of bicarbonate between 24 and 48 h after admission to the ICU. Δbicarbonate = bicarbonate T0—bicarbonate T1. Bicarbonate T0 was divided into ≤ Q_1_, Q_1_-Q_2_, Q_2_-Q_3_, and >Q_3_ groups according to the quartiles, and Q_1_ was 21 mEq/L, Q_2_ was 23 mEq/L, and Q_3_ was 26 mEq/L. Δbicarbonate was also divided into ≤ Q_1_, Q_1_-Q_2_, Q_2_-Q_3_, and >Q_3_ groups according to the quartiles, and Q_1_ was −2 mEq/L, Q_2_ was 0 mEq/L, and Q_3_ was 2 mEq/L.

Whether the participant survived within 30 days was regarded as the outcome of this study. In-hospital mortality was recorded by the MIMIC III and MIMIC IV databases, while out-of-hospital mortality was recorded by the Social Security Bureau. The median follow-up time was 30.00 (30.00, 30.00) days.

### Statistical analysis

The mean ± standard deviation (SD) was used to describe the measurement data with a normal distribution, and a *t*-test was applied to compare the difference between the two groups. The median and quartile [M (Q_1_, Q_3_)] were used to display the measurement data with a non-normal distribution, and the Wilcoxon rank sum test was employed to compare the difference between the two groups. The enumeration data were shown as the number of cases and the component ratio [*n* (%)], and differences between groups were compared using the chi-squared test or Fisher's exact probability methods. Variables with missing values ≥20% were deleted. The Random Forest interpolation method (n_estimators = 500) was applied to variables with missing values of < 20% (Supplementary Table 1), and sensitivity analysis was performed to compare the data before and after interpolation (Supplementary Table 2). Confounders were identified using the univariate Cox proportional risk models, which were then subjected to stepwise regression analysis. Univariate and multivariable Cox proportional risk models were utilized for exploring the relationship between bicarbonate T0 and Δbicarbonate with 30-day mortality in patients with acute ischemic stroke. To explore the association between bicarbonate T0 and 30-day mortality in patients with acute ischemic stroke, a multivariable model was adjusted for age, gender, ethnicity, ventilation, vasopressor, hyperlipidemia, atrial fibrillation, heart rate, hemoglobin, RDW, CCI, SAPSII, CABG, thrombolysis, antiplatelet agents, and anticoagulation agents. To explore the association between Δbicarbonate and 30-day mortality in patients with acute ischemic stroke, a multivariable model was adjusted for age, gender, ethnicity, ventilation, vasopressor, hyperlipidemia, atrial fibrillation, diastolic, RDW, glucose, CCI, SAPSII, CABG, thrombolysis, antiplatelet agents, and anticoagulation agents. A sensitivity analysis was conducted by comparing the data before and after interpolating the missing data. A subgroup analysis was conducted by stratifying age, CCI, thrombolytic therapy, antiplatelet therapy, and anticoagulant therapy. The Kaplan–Meier curves were plotted to measure the 30-day survival probability of patients with acute ischemic stroke. The Hazard Ratio (HR) was applied to evaluate the associations between bicarbonate T0 and Δbicarbonate with 30-day mortality in patients with acute ischemic stroke. The value of alpha equal to 0.05 was set as the confidence level. Missing value interpolation was completed using Python 3.7.4 (Python Software Foundation, Delaware, USA). Sensitivity analysis, difference comparison, univariate/multivariate Cox proportional risk model modeling, and subgroup analysis were performed using SAS 9.4 (SAS Institute Inc., Cary, NC, USA). The Kaplan–Meier curve was drawn using R version 4.2.0 (2022-04-22 ucrt).

## Results

### Comparisons of the characteristics of acute ischemic stroke patients between the survival and death groups

**Figure 1:**
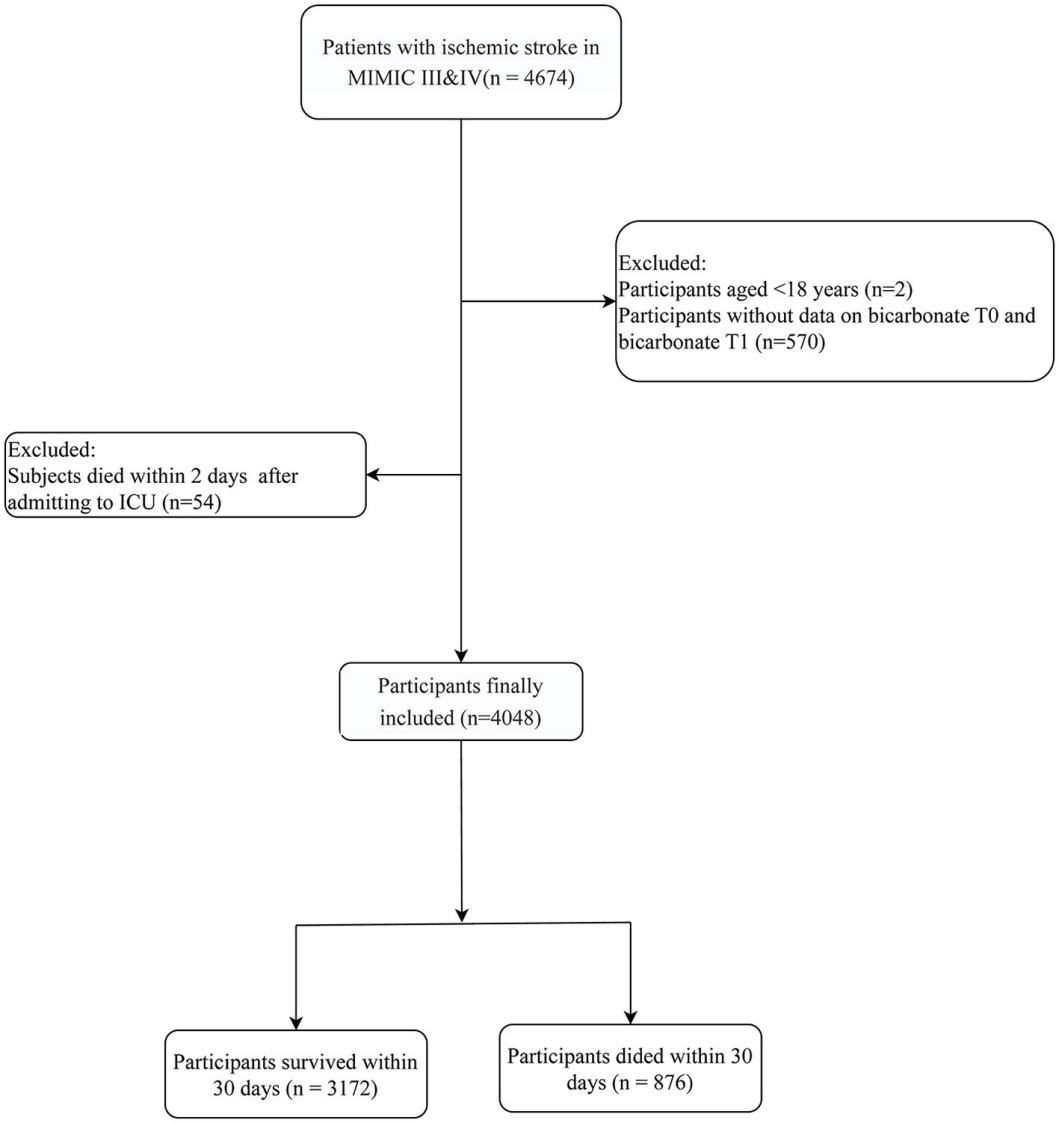
The flow chart selecting the participants in the current study.

This study collected data from both the MIMIC-III and MIMIC-IV databases, which included information from 4,674 participants with acute ischemic stroke. Among them, people aged < 18 years (*n* = 2), without measurements on bicarbonate levels at 24-h intervals (*n* = 570), and who died within 2 days of being admitted to the ICU (*n* = 54) were excluded. Finally, 4,048 participants were included. The screening process is shown in Figure 1.

Compared with the survival group, the mean age (73.29 vs. 66.14 years), respiratory rate (19.41 beats vs. 18.41 beats), heart rate (86.46 beats vs. 82.67 beats), and RDW (15.05 vs. 14.39%) in the death group were high. The percentages of patients who received ventilation (83.79 vs. 61.38%) and vasopressors (43.84 vs. 28.31%) in the death group were higher than those in the percentages of the survival group. The percentages of participants who received thrombolysis (10.16 vs. 14.28%), antiplatelet agents (55.14 vs. 62.30%), and anticoagulation agents (9.70 vs. 22.35%) in the death group were lower than those in the survival group. The median length of stay (LOS) in the death group was longer than the survival group (5.02 vs. 3.07 days) (Table 1).

**Table 1:** Comparisons of characteristics of acute ischemic stroke patients the between survival and death groups.

		**Group**			
**Variables**	**Total (*****n*** = **4,048)**	**Survival group (*****n*** = **3,172)**	**Death group (*****n*** = **876)**	**Statistics**	***P***
Age, years, Mean ± SD	67.93 ± 15.57	66.44 ± 15.76	73.29 ± 13.58	*t* = −12.75	< 0.001
Gender, *n* (%)				χ^2^ = 0.816	0.366
Female	2,034 (50.25)	1,582 (49.87)	452 (51.60)		
Male	2,014 (49.75)	1,590 (50.13)	424 (48.40)		
Ethnicity, *n* (%)				χ^2^ = 36.357	< 0.001
Black	423 (10.45)	352 (11.10)	71 (8.11)		
Others	480 (11.86)	388 (12.23)	92 (10.50)		
Unknown	496 (12.25)	340 (10.72)	156 (17.81)		
White	2,649 (65.44)	2,092 (65.95)	557 (63.58)		
Ventilation, n (%)				χ^2^ = 154.121	< 0.001
No	1,367 (33.77)	1,225 (38.62)	142 (16.21)		
Yes	2,681 (66.23)	1,947 (61.38)	734 (83.79)		
Vasopressor, *n* (%)				χ^2^ = 76.458	< 0.001
No	2,766 (68.33)	2,274 (71.69)	492 (56.16)		
Yes	1,282 (31.67)	898 (28.31)	384 (43.84)		
CABG, *n* (%)				χ^2^ = 7.355	0.007
No	3,958 (97.78)	3,091 (97.45)	867 (98.97)		
Yes	90 (2.22)	81 (2.55)	9 (1.03)		
Coronary artery disease, n (%)				χ^2^ = 14.886	< 0.001
No	2,768 (68.38)	2,216 (69.86)	552 (63.01)		
Yes	1,280 (31.62)	956 (30.14)	324 (36.99)		
Congestive heart failure, *n* (%)				χ^2^ = 33.660	< 0.001
No	2,972 (73.42)	2,396 (75.54)	576 (65.75)		
Yes	1,076 (26.58)	776 (24.46)	300 (34.25)		
Peripheral vascular disease, *n* (%)				χ^2^ = 0.665	0.415
No	3,468 (85.67)	2,725 (85.91)	743 (84.82)		
Yes	580 (14.33)	447 (14.09)	133 (15.18)		
Hyperlipidemia, *n* (%)				χ^2^ = 14.048	< 0.001
No	2,200 (54.35)	1,675 (52.81)	525 (59.93)		
Yes	1,848 (45.65)	1,497 (47.19)	351 (40.07)		
Hypertension, *n* (%)				χ^2^ = 0.004	0.949
No	2,282 (56.37)	1,789 (56.40)	493 (56.28)		
Yes	1,766 (43.63)	1,383 (43.60)	383 (43.72)		
Chronic kidney disease, *n* (%)				χ^2^ = 26.655	< 0.001
No	3,375 (83.37)	2,695 (84.96)	680 (77.63)		
Yes	673 (16.63)	477 (15.04)	196 (22.37)		
Atrial fibrillation, *n* (%)				χ^2^ = 29.072	< 0.001
No	2,710 (66.95)	2,190 (69.04)	520 (59.36)		
Yes	1,338 (33.05)	982 (30.96)	356 (40.64)		
Malignant cancer, *n* (%)				χ^2^ = 69.130	< 0.001
No	3,708 (91.60)	2,966 (93.51)	742 (84.70)		
Yes	340 (8.40)	206 (6.49)	134 (15.30)		
Diabetes, *n* (%)				χ^2^ = 1.198	0.274
No	2,733 (67.51)	2,155 (67.94)	578 (65.98)		
Yes	1,315 (32.49)	1,017 (32.06)	298 (34.02)		
Systolic blood pressure, mmHg, Mean ± SD	137.44 ± 27.15	138.38 ± 26.54	134.06 ± 29.01	*T* = 3.97	< 0.001
Diastolic blood pressure, mmHg, Mean ± SD	72.72 ± 18.77	73.15 ± 18.39	71.13 ± 20.02	*t* = 2.70	0.007
Respiratory rate, bpm, Mean ± SD	18.63 ± 5.00	18.41 ± 4.84	19.42 ± 5.49	*t* = −4.94	< 0.001
Heart rate, bpm, Mean ± SD	83.64 ± 18.27	82.73 ± 17.62	86.95 ± 20.13	*t* = −5.64	< 0.001
Temperature, °C, Mean ± SD	36.72 ± 0.81	36.73 ± 0.75	36.70 ± 0.99	*t* = 0.80	0.424
SPO_2_, %, Mean ± SD	97.62 ± 2.58	97.62 ± 2.52	97.64 ± 2.82	*t* = −0.20	0.840
WBC, K/uL, M (Q_1_, Q_3_)	10.40 (8.00, 13.70)	10.10 (7.80, 13.40)	11.50 (8.80, 15.50)	Z = 7.026	< 0.001
Platelet, K/uL, M (Q_1_, Q_3_)	209.00 (158.00, 268.50)	209.00 (162.00, 266.50)	207.00 (149.00, 275.00)	Z = −1.557	0.119
Hemoglobin, g/dL, Mean ± SD	11.54 ± 2.26	11.66 ± 2.26	11.11 ± 2.21	*t* = 6.31	< 0.001
RDW, %, Mean ± SD	14.53 ± 1.96	14.39 ± 1.91	15.05 ± 2.05	*t* = −8.53	< 0.001
BUN, mg/dL, M (Q_1_, Q_3_)	18.00 (13.00, 26.00)	17.00 (12.00, 24.00)	22.00 (15.00, 35.00)	Z = 12.311	< 0.001
Creatinine, mg/dL, M (Q_1_, Q_3_)	0.90 (0.70, 1.28)	0.90 (0.70, 1.20)	1.00 (0.80, 1.50)	Z = 7.807	< 0.001
Glucose, mg/dL, M (Q_1_, Q_3_)	128.00 (105.00, 164.00)	125.00 (104.00, 158.00)	139.00 (114.00, 184.00)	Z = 8.448	< 0.001
CCI score, M (Q_1_, Q_3_)	5.00 (3.00, 7.00)	4.00 (3.00, 6.00)	6.00 (4.00, 8.00)	Z = 11.257	< 0.001
SAPSII score, M (Q_1_, Q_3_)	33.00 (26.00, 42.00)	31.00 (24.00, 40.00)	42.00 (35.00, 51.00)	Z = 21.765	< 0.001
SOFA score, M (Q_1_, Q_3_)	4.00 (2.00, 6.00)	3.00 (2.00, 5.00)	5.00 (3.00, 8.00)	Z = 15.479	< 0.001
qSOFA score, M (Q_1_, Q_3_)	2.00 (1.00, 2.00)	2.00 (1.00, 2.00)	2.00 (2.00, 3.00)	Z = 9.703	< 0.001
SIRS score, M (Q_1_, Q_3_)	1.00 (0.00, 1.00)	1.00 (0.00, 1.00)	1.00 (0.00, 2.00)	Z = 9.183	< 0.001
Thrombolysis, *n* (%)				χ^2^ = 10.054	0.002
No	3,506 (86.61)	2,719 (85.72)	787 (89.84)		
Yes	542 (13.39)	453 (14.28)	89 (10.16)		
Antiplatelet agents, *n* (%)				χ^2^ = 14.750	< 0.001
No	1,589 (39.25)	1,196 (37.70)	393 (44.86)		
Yes	2,459 (60.75)	1,976 (62.30)	483 (55.14)		
Anticoagulation agents, *n* (%)				χ^2^ = 69.651	< 0.001
No	3,254 (80.39)	2,463 (77.65)	791 (90.30)		
Yes	794 (19.61)	709 (22.35)	85 (9.70)		
LOS, days, M (Q_1_, Q_3_)	3.57 (1.84, 7.51)	3.07 (1.70, 7.06)	5.02 (2.94, 8.78)	Z = 10.498	< 0.001
Follow-up time, days, M (Q_1_, Q_3_)	30.00 (30.00, 30.00)	30.00 (30.00, 30.00)	8.69 (5.11, 15.95)	Z = −62.992	< 0.001
Bicarbonate T0, mEq/L, Mean ± SD	23.24 ± 3.90	23.48 ± 3.67	22.36 ± 4.55	*t* = 6.72	< 0.001
Bicarbonate T0, *n* (%)				χ^2^ = 76.501	< 0.001
≤ Q1	836 (20.65)	570 (17.97)	266 (30.37)		
Q1–Q2	757 (18.70)	581 (18.32)	176 (20.09)		
Q2–Q3	1,406 (34.73)	1,170 (36.89)	236 (26.94)		
>Q3	1,049 (25.91)	851 (26.83)	198 (22.60)		
Bicarbonate T1, mEq/L, Mean ± SD	23.62 ± 3.77	23.95 ± 3.59	22.43 ± 4.13	*t* = 9.96	< 0.001
Bicarbonate T1, *n* (%)				χ^2^ = 122.989	< 0.001
≤ Q_1_	762 (18.82)	493 (15.54)	269 (30.71)		
Q_1_-Q_2_	1,198 (29.59)	927 (29.22)	271 (30.94)		
Q_2_-Q_3_	896 (22.13)	745 (23.49)	151 (17.24)		
>Q_3_	1,192 (29.45)	1,007 (31.75)	185 (21.12)		
ΔBicarbonate, M (Q_1_, Q_3_)	0.00 (-2.00, 2.00)	0.00 (-2.00, 1.00)	0.00 (-2.00, 2.00)	Z = 3.315	< 0.001
ΔBicarbonate, *n* (%)				χ^2^ = 9.785	0.020
≤ Q_1_	874 (21.59)	712 (22.45)	162 (18.49)		
Q_1_-Q_2_	1,010 (24.95)	793 (25.00)	217 (24.77)		
Q_2_-Q_3_	1,116 (27.57)	875 (27.59)	241 (27.51)		
>Q_3_	1,048 (25.89)	792 (24.97)	256 (29.22)		
Mechanical bolt removal, *n* (%)				χ^2^ = 0.675	0.411
No	3,935 (97.21)	3,087 (97.32)	848 (96.80)		
Yes	113 (2.79)	85 (2.68)	28 (3.20)		
Hemorrhagic transformation, *n* (%)				χ^2^ = 0.594	0.441
No	3,344 (82.61)	2,628 (82.85)	716 (81.74)		
Yes	704 (17.39)	544 (17.15)	160 (18.26)		

SD, standard deviation; M, Median; Q_1_,1st Quartile; Q_3_, 3st Quartile; CABG, coronary artery bypass grafting; SPO_2_, oxygen saturation; WBC, white blood cell; RDW, red cell distribution width; BUN, blood urea nitrogen; CCI, Charlson comorbidity index; SAPSII, simplified acute physiology score II; SOFA, sequential organ failure assessment; qSOFA, quick sequential organ failure assessment; SIRS, systemic inflammatory response syndrome; LOS, length of stay.

### Potential confounders for the associations of bicarbonate T0 and Δbicarbonate with 30-day mortality in patients with acute ischemic stroke

To identify the associations between bicarbonate T0 and Δbicarbonate and 30-day mortality in patients with acute ischemic stroke, potential confounders were found using univariate Cox proportional hazard model analysis. The data revealed that several factors, including age, being Black, ventilation, vasopressor use, coronary artery disease, hyperlipidemia, chronic kidney disease, atrial fibrillation, systolic blood pressure, diastolic blood pressure, respiratory rate, heart rate, WBC count, hemoglobin levels, RDW, BUN, creatinine, glucose levels, CCI, SAPS II, SOFA, qSOFA, SIRS, CABG, thrombolysis, antiplatelet agents, and anticoagulation agents, might be potential confounding variables in the associations between bicarbonate T0 and Δbicarbonate levels and 30-day mortality risk in patients with acute ischemic stroke (Table 2).

**Table 2:** Potential confounding factors associated with 30-day mortality in patients with acute ischemic stroke.

**Variables**	**β**	**S.E**	**χ^2^**	**HR (95%CI)**	***P***
Age	0.029	0.003	129.201	1.03 (1.02–1.03)	< 0.001
Gender					
Female				Ref	
Male	−0.068	0.068	1.005	0.93 (0.82–1.07)	0.316
Ethnicity					
Black	−0.264	0.126	4.379	0.77 (0.60–0.98)	0.036
Others	−0.100	0.113	0.794	0.90 (0.73–1.13)	0.373
Unknown	0.472	0.091	27.147	1.60 (1.34–1.91)	< 0.001
White				Ref	
Ventilation					
No				Ref	
Yes	1.078	0.092	138.168	2.94 (2.46–3.52)	< 0.001
Vasopressor					
No				Ref	
Yes	0.602	0.068	78.051	1.83 (1.60–2.09)	< 0.001
Coronary artery disease					
No				Ref	
Yes	0.262	0.070	13.982	1.30 (1.13–1.49)	< 0.001
Hyperlipidemia					
No				Ref	
Yes	−0.252	0.069	13.318	0.78 (0.68–0.89)	< 0.001
Hypertension					
No				Ref	
Ye	0.005	0.068	0.005	1.00 (0.88–1.15)	0.943
Chronic kidney disease					
No				Ref	
Yes	0.398	0.081	24.110	1.49 (1.27–1.75)	< 0.001
Atrial fibrillation					
No				Ref	
Yes	0.375	0.069	29.746	1.46 (1.27–1.67)	< 0.001
Systolic blood pressure	−0.005	0.001	17.810	0.99 (0.99–0.99)	< 0.001
Diastolic blood pressure	−0.005	0.002	8.615	0.99 (0.99–0.99)	0.003
Respiratory rate	0.034	0.006	27.586	1.03 (1.02–1.05)	< 0.001
Heart rate	0.011	0.002	37.143	1.01 (1.01–1.01)	< 0.001
Temperature	−0.043	0.043	0.981	0.96 (0.88–1.04)	0.322
SPO_2_	0.004	0.013	0.109	1.00 (0.98–1.03)	0.741
WBC	0.010	0.002	23.159	1.01 (1.01–1.01)	< 0.001
Platelet	−0.000	0.000	1.298	1.00 (1.00–1.00)	0.255
Hemoglobin	−0.089	0.015	36.690	0.91 (0.89–0.94)	< 0.001
RDW	0.116	0.013	74.577	1.12 (1.09–1.15)	< 0.001
BUN	0.015	0.001	123.461	1.01 (1.01–1.02)	< 0.001
Creatinine	0.060	0.013	19.737	1.06 (1.03–1.09)	< 0.001
Glucose	0.002	0.000	58.403	1.01 (1.01–1.01)	< 0.001
CCI score	0.141	0.012	148.302	1.15 (1.13–1.18)	< 0.001
SAPSII score	0.049	0.002	518.122	1.05 (1.05–1.05)	< 0.001
SOFA score	0.120	0.008	235.215	1.13 (1.11–1.15)	< 0.001
qSOFA score	0.414	0.044	87.047	1.51 (1.39–1.65)	< 0.001
SIRS score	0.358	0.038	86.698	1.43 (1.33–1.54)	< 0.001
CABG					
No				Ref	
Yes	−0.857	0.335	6.550	0.42 (0.22–0.82)	0.010
Thrombolysis					
No				Ref	
Yes	−0.346	0.112	9.561	0.71 (0.57–0.88)	0.002
Antiplatelet agents					
No				Ref	
Yes	−0.265	0.068	15.219	0.77 (0.67–0.88)	< 0.001
Anticoagulation agents					
No				Ref	
Yes	−0.918	0.114	64.733	0.40 (0.32–0.50)	< 0.001
Los	0.004	0.004	0.787	1.00 (1.00–1.01)	0.375
Mechanical bolt removal					
No				Ref	
Yes	0.171	0.192	0.789	1.19 (0.81–1.73)	0.375
Hemorrhagic transformation					
No				Ref	
Yes	0.056	0.087	0.415	1.06 (0.89–1.26)	0.519

Ref, Reference; HR, Hazard Ratio; CI, Confidence Interval; SPO_2_, oxygen saturation; WBC, white blood cell; RDW, red cell distribution width; BUN, blood urea nitrogen; CCI, Charlson comorbidity index; SAPSII, simplified acute physiology score II; SOFA, sequential organ failure assessment; qSOFA, quick sequential organ failure assessment; SIRS, systemic inflammatory response syndrome; CABG, coronary artery bypass grafting; LOS, length of stay.

### Associations of bicarbonate T0 with 30-day mortality in patients with acute ischemic stroke

**Figure 2:**
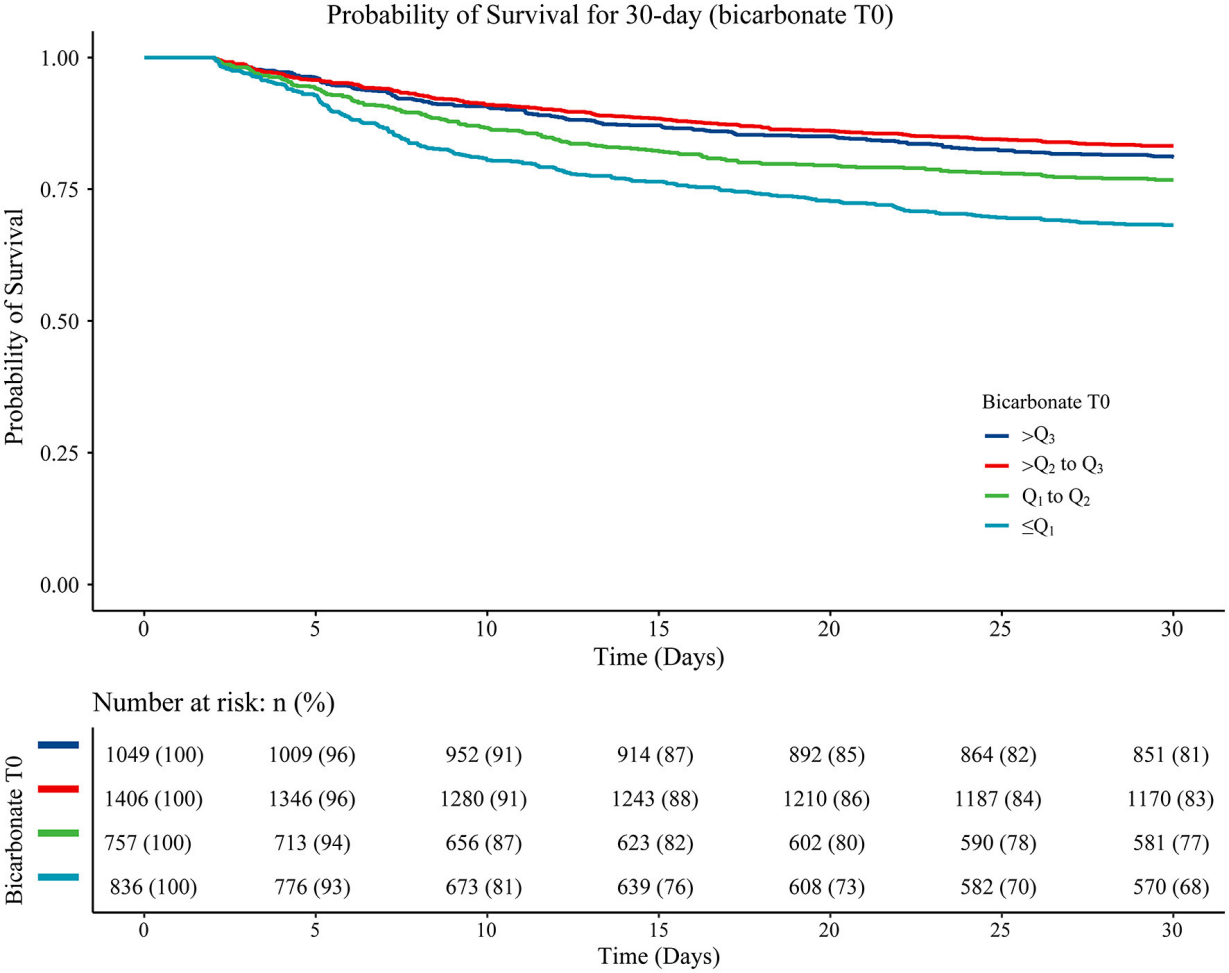
The Kaplan–Meier curve showing the 30-day survival probability in patients from different bicarbonate T0 group. Log-rank ≤ Q_1_ vs. Q_1_-Q_2_ (*P* < 0.001), ≤ Q_1_ vs. Q_2_-Q_3_ (*P* < 0.001), ≤ Q_1_ vs. >Q_3_ (*P* < 0.001), Q_1_-Q_2_ vs. Q_2_-Q_3_ (*P* = 0.022), Q_1_-Q_2_ vs. >Q_3_ (*P* < 0.001), and Q_2_-Q_3_ vs. >Q_3_ (*P* = 0.185).

Variables with a statistically significant difference were involved in the multivariable Cox proportional hazard model through stepwise regression. The results are displayed in Table 3, with age (HR = 1.02, 95%CI: 1.02–1.03), being Black (HR = 0.75, 95%CI: 0.59–0.97), ventilation (HR = 2.16, 95%CI: 1.78–2.62), vasopressor use (HR = 1.27, 95%CI: 1.09–1.48), hyperlipidemia (HR = 0.80, 95%CI: 0.70–0.92), atrial fibrillation (HR = 1.22, 95%CI: 1.05–1.41), diastolic blood pressure (HR = 1.01, 95%CI: 1.01–1.01), heart rate (HR = 1.01, 95%CI: 1.01–1.01), RDW (HR = 1.05, 95%CI: 1.01–1.08), CCI (HR = 1.09, 95%CI: 1.06–1.12), SAPS II (HR = 1.03, 95%CI: 1.02–1.04), CABG (HR = 0.22, 95%CI: 0.11–0.43), thrombolysis (HR = 0.72, 95%CI: 0.58–0.90), antiplatelet agents (HR = 0.82, 95%CI: 0.72–0.95), and anticoagulation agents (HR = 0.35, 95%CI: 0.28–0.44) considered as confounders associated with 30-day mortality in patients with acute ischemic stroke. Gender was an important variable associated with mortality in patients with acute ischemic stroke, which was also adjusted for in the multivariable Cox proportional hazard model. The data revealed that after adjusting for these confounding factors, bicarbonate T0 ≤ Q_1_ (HR = 1.22, 95%CI: 1.01–1.48) or bicarbonate T0 of Q_1_-Q_2_ (HR = 1.26, 95%CI: 1.03–1.55) was associated with an increased risk of 30-day mortality in patients with acute ischemic stroke compared with bicarbonate T0 >Q_3_ (Table 3). The 30-day survival probability of acute ischemic stroke patients with bicarbonate T0 of Q_2_-Q_3_, bicarbonate T0 of Q_2_-Q_3_, or bicarbonate T0 >Q_3_ was higher than that of the participants with bicarbonate T0 ≤ Q_1_ (Figure 2).

**Table 3:** Association between bicarbonate T0 and 30-day mortality in patients with acute ischemic stroke.

**Variables**	**β**	**S.E**	**χ^2^**	**HR (95%CI)**	***P***
Bicarbonate T0					
≤ Q_1_	0.202	0.098	4.267	1.22 (1.01–1.48)	0.039
Q_1_-Q_2_	0.234	0.105	4.977	1.26 (1.03–1.55)	0.026
Q_2_-Q_3_	−0.048	0.097	0.242	0.95 (0.79–1.15)	0.623
>Q_3_				Ref	
Age	0.023	0.003	58.877	1.02 (1.02–1.03)	< 0.001
Gender					
Female				Ref	
Male	0.070	0.070	1.003	1.07 (0.94–1.23)	0.316
Ethnicity					
Black	−0.282	0.129	4.755	0.75 (0.59–0.97)	0.029
Others	0.053	0.114	0.214	1.05 (0.84–1.32)	0.644
Unknown	0.521	0.093	31.485	1.68 (1.40–2.02)	< 0.001
White				Ref	
Ventilation					
No				Ref	
Yes	0.772	0.098	61.548	2.16 (1.78–2.62)	< 0.001
Vasopressor					
No				Ref	
Yes	0.240	0.079	9.347	1.27 (1.09–1.48)	0.002
Hyperlipidemia					
No				Ref	
Yes	−0.217	0.071	9.408	0.80 (0.70–0.92)	0.002
Atrial fibrillation					
No				Ref	
Yes	0.196	0.074	6.953	1.22 (1.05–1.41)	0.008
Diastolic blood pressure	0.004	0.002	6.136	1.01 (1.01–1.01)	0.013
Heart rate	0.004	0.002	4.626	1.01 (1.01–1.01)	0.031
RDW	0.045	0.016	8.435	1.05 (1.01–1.08)	0.004
CCI score	0.088	0.013	44.732	1.09 (1.06–1.12)	< 0.001
SAPSII score	0.030	0.003	100.206	1.03 (1.02–1.04)	< 0.001
CABG					
No				Ref	
Ye	−1.507	0.341	19.505	0.22 (0.11–0.43)	< 0.001
Thrombolysis					
No				Ref	
Yes	−0.325	0.114	8.150	0.72 (0.58–0.90)	0.004
Antiplatelet agents					
No				Ref	
Yes	−0.193	0.070	7.541	0.82 (0.72–0.95)	0.006
Anticoagulation agents					
No				Ref	
Yes	−1.049	0.117	79.834	0.35 (0.28–0.44)	< 0.001

Ref, Reference; HR, Hazard Ratio; CI, Confidence Interval; RDW, red cell distribution width; CCI, Charlson comorbidity index; SAPSII, simplified acute physiology score II; CABG, coronary artery bypass grafting.

When bicarbonate T0 was dealt with as a continuous variable, we found that age (HR = 1.02, 95%CI: 1.02–1.03), Black (HR = 0.75, 95%CI: 0.58–0.97), ventilation (HR = 2.19, 95%CI: 1.80–2.65), vasopressor (HR = 1.27, 95%CI: 1.09–1.49), hyperlipidemia (HR = 0.80, 95%CI: 0.70–0.92), atrial fibrillation (HR = 1.22, 95%CI: 1.05–1.41), diastolic blood pressure (HR = 1.01, 95%CI: 1.01–1.01), heart rate (HR = 1.01, 95%CI: 1.01–1.01), RDW (HR = 1.05, 95%CI: 1.02–1.08), CCI (HR = 1.09, 95%CI: 1.06–1.12), SAPS II (HR = 1.03, 95%CI: 1.02–1.04), CABG (HR = 0.23, 95%CI: 0.12–0.44), thrombolysis (HR = 0.72, 95%CI: 0.57–0.90), antiplatelet agents (HR = 0.83, 95%CI: 0.72–0.95), and anticoagulation agents (HR = 0.35, 95%CI: 0.28–0.45) were confounding factors. After adjusting for these variables and gender, increased bicarbonate T0 was related to a decreased risk of 30-day mortality in patients with acute ischemic stroke (HR = 0.98, 95%CI: 0.97–0.99) (Supplementary Table 3).

### Relationship between Δbicarbonate with 30-day mortality in patients with acute ischemic stroke

**Figure 3:**
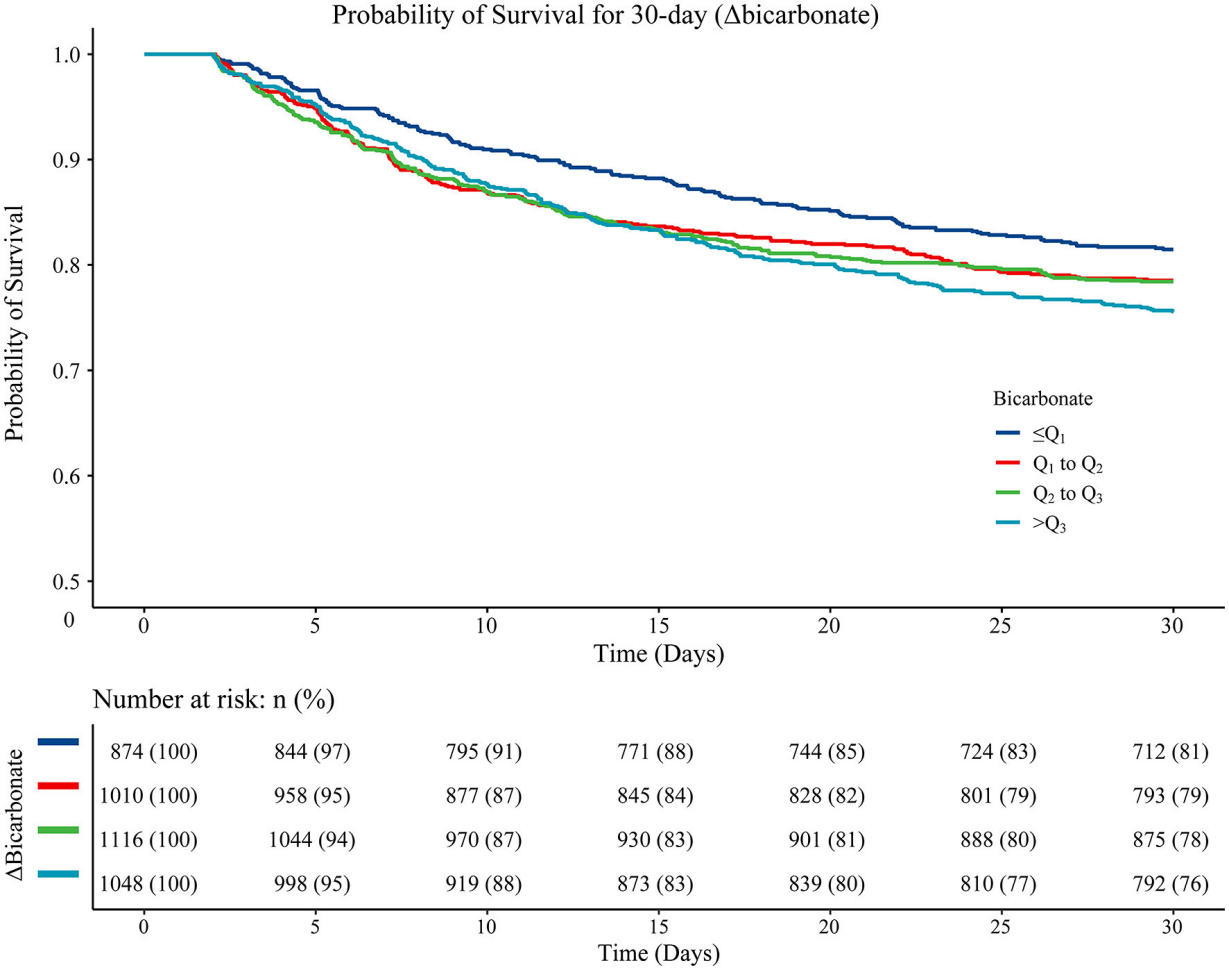
The Kaplan–Meier curve presenting the 30-day survival probability in patients from different Δbicarbonate group. Log-rank ≤ Q_1_ vs. Q_1_-Q_2_ (*P* = 0.160), ≤ Q_1_ vs. Q_2_-Q_3_ (*P* = 0.160), ≤ Q_1_ vs. >Q_3_ (*P* = 0.010), Q_1_-Q_2_ vs. Q_2_-Q_3_ (*P* = 0.930), Q_1_-Q_2_ vs. >Q_3_ (*P* = 0.210), and Q_2_-Q_3_ vs. >Q_3_ (*P* = 0.210).

As for the association between Δbicarbonate and 30-day mortality in acute ischemic stroke patients, the results of the multivariable Cox proportional hazard model revealed that age (HR = 1.02, 95%CI: 1.02–1.03), being Black (HR = 0.75, 95%CI: 0.58–0.97), ventilation (HR = 2.20, 95%CI: 1.81–2.66), vasopressor use (HR = 1.33, 95%CI: 1.14–1.55), hyperlipidemia (HR = 0.81, 95%CI: 0.70–0.93), atrial fibrillation (HR = 1.21, 95%CI: 1.05–1.40), diastolic blood pressure (HR = 1.01, 95%CI: 1.01–1.01), heart rate (HR = 1.01, 95%CI: 1.01–1.01), RDW (HR = 1.05, 95%CI: 1.02–1.08), CCI (HR = 1.09, 95%CI: 1.06–1.12), SAPSII (HR = 1.03, 95%CI: 1.03–1.04), CABG (HR = 0.23, 95%CI: 0.12–0.45), thrombolysis (HR = 0.70, 95%CI: 0.56–0.88), antiplatelet agents (HR = 0.82, 95%CI: 0.72–0.94), and anticoagulation agents (HR = 0.35, 95%CI: 0.28–0.44) were confounding factors associated with the mortality in acute ischemic stroke patients. In the multivariable Cox proportional hazard model, Δbicarbonate of Q_1_-Q_2_ (HR = 1.36, 95%CI: 1.11–1.67), Δbicarbonate of Q_2_-Q_3_ (HR = 1.40, 95%CI: 1.14–1.71), and Δbicarbonate >Q_3_ (HR = 1.37, 95%CI: 1.12–1.03) were correlated with an elevated risk of 30-day mortality in acute ischemic stroke patients (Table 4). The Kaplan–Meier curve showed that the 30-day survival probability in patients with Δbicarbonate ≤ Q_1_ group was higher than that in patients with Δbicarbonate >Q_3_ group (Figure 3).

**Table 4:** Association between Δbicarbonate and 30-day mortality in patients with acute ischemic stroke.

**Variables**	**β**	**S.E**.	**χ^2^**	**HR (95%CI)**	***P***
Δbicarbonate					
≤ Q_1_				Ref	
Q_1_ to Q_2_	0.307	0.105	8.629	1.36 (1.11–1.67)	0.003
Q_2_ to Q_3_	0.333	0.103	10.520	1.40 (1.14–1.71)	0.001
>Q_3_	0.312	0.101	9.503	1.37 (1.12–1.67)	0.002
Age	0.022	0.003	54.807	1.02 (1.02–1.03)	< 0.001
Gender					
Female				Ref	
Male	0.063	0.070	0.827	1.07 (0.93–1.22)	0.363
Ethnicity					
Black	−0.287	0.129	4.923	0.75 (0.58–0.97)	0.027
Others	0.095	0.114	0.686	1.10 (0.88–1.38)	0.407
Unknown	0.531	0.093	32.874	1.70 (1.42–2.04)	< 0.001
White				Ref	
Ventilation					
No				Ref	
Yes	0.788	0.098	64.306	2.20 (1.81–2.67)	< 0.001
Vasopressor					
No				Ref	
Yes	0.286	0.078	13.365	1.33 (1.14–1.55)	< 0.001
Hyperlipidemia					
No				Ref	
Yes	−0.217	0.071	9.299	0.81 (0.70–0.93)	0.002
Atrial fibrillation					
No				Ref	
Yes	0.193	0.074	6.767	1.21 (1.05–1.40)	0.009
Diastolic blood pressure	0.004	0.002	6.137	1.01 (1.01–1.01)	0.013
Heart rate	0.004	0.002	4.161	1.01 (1.01–1.01)	0.041
RDW	0.048	0.016	9.374	1.05 (1.02–1.08)	0.002
CCI score	0.085	0.013	41.588	1.09 (1.06–1.12)	< 0.001
SAPSII score	0.032	0.003	120.319	1.03 (1.03–1.04)	< 0.001
CABG					
No				Ref	
Yes	−1.466	0.340	18.538	0.23 (0.12–0.45)	< 0.001
Thrombolysis					
No				Ref	
Yes	−0.350	0.114	9.444	0.70 (0.56–0.88)	0.002
Antiplatelet agents					
No				Ref	
Yes	−0.198	0.070	7.990	0.82 (0.72–0.94)	0.005
Anticoagulation agents					
No				Ref	
Yes	−1.045	0.117	79.460	0.35 (0.28–0.44)	< 0.001

Ref, Reference; HR, Hazard Ratio; CI, Confidence Interval; RDW, red cell distribution width; CCI, Charlson comorbidity index; SAPSII, simplified acute physiology score II; CABG, coronary artery bypass grafting.

When Δbicarbonate was considered a continuous variable, an increased risk of 30-day mortality in patients with acute ischemic stroke was identified in those with higher Δbicarbonate (HR = 1.03, 95%CI: 1.01–1.05) after adjusting for confounding factors, including age (HR = 1.02, 95%CI: 1.02–1.03), gender, ethnicity (HR = 0.75, 95%CI: 0.59–0.97), ventilation (HR = 2.20, 95%CI: 1.82–2.67), vasopressor (HR = 1.31, 95%CI: 1.13–1.53), hyperlipidemia (HR = 0.81, 95%CI: 0.71–0.93), atrial fibrillation (HR = 1.22, 95%CI: 1.05–1.41), diastolic blood pressure (HR = 1.01, 95%CI: 1.01–1.01), heart rate (HR = 1.01, 95%CI: 1.01–1.01), RDW (HR = 1.05, 95%CI: 1.02–1.08), CCI (HR = 1.09, 95%CI: 1.06–1.12), SAPSII (HR = 1.03, 95%CI: 1.03–1.04), CABG (HR = 0.23, 95%CI: 0.12–0.44), thrombolysis (HR = 0.70, 95%CI: 0.56–0.88), antiplatelet agents (HR = 0.82, 95%CI: 0.71–0.94), and anticoagulation agents (HR = 0.35, 95%CI: 0.28–0.44) (Supplementary Table 4).

### Subgroup analysis of associations of bicarbonate T0 and Δbicarbonate with 30-day mortality in patients with acute ischemic stroke

In patients aged < 70 years, bicarbonate T0 of Q_2_-Q_3_ was associated with an increased risk of 30-day mortality in patients with acute ischemic stroke in the adjusted model (HR = 1.67, 95%CI: 1.18–2.37). In people aged ≥70 years, bicarbonate T0 ≤ Q_1_ was linked with an elevated risk of 30-day mortality in patients with acute ischemic stroke (HR = 1.28, 95%CI: 1.01–1.61). Higher bicarbonate T0 was related to an increased risk of 30-day mortality in patients with acute ischemic stroke, which was observed in those aged ≥70 years (HR = 1.05, 95%CI: 1.02–1.08). After adjusting for potential confounders, we found that subjects withΔbicarbonate of Q_1_-Q_2_ (HR =1.56, 95%CI: 1.19–2.04), Δbicarbonate of Q_2_-Q_3_ (HR = 1.58, 95%CI: 1.21–2.06), and Δbicarbonate >Q_3_ (HR = 1.65, 95%CI: 1.28–2.14) were associated with an increased risk of 30-day mortality in patients with acute ischemic stroke in patients aged ≥70 years. In those with CCI≥5, Δbicarbonate of Q_1_-Q_2_ (HR = 1.57, 95%CI: 1.20–2.06), Δbicarbonate of Q_2_-Q_3_ (HR = 1.63, 95%CI: 1.25–2.12), or Δbicarbonate>Q_3_ (HR = 1.66, 95%CI: 1.29–2.15) were correlated with an increased risk of 30-day mortality in patients with acute ischemic stroke. An increased risk of 30-day mortality in patients with acute ischemic stroke was elevated with the increase of Δbicarbonate (HR = 1.05, 95%CI: 1.02–1.07) in people with CCI ≥ 5 (Table 5).

**Table 5:** Subgroup analysis of associations of bicarbonate T0 and Δbicarbonate with 30-day mortality in patients with acute ischemic stroke.

**Subgroups**	**HR (95%CI)**	***P***
Age < 70 years (*n* = 2,031)		
Bicarbonate T0		
≤ Q_1_	1.12 (0.80–1.57)	0.506
Q_1_-Q_2_	1.67 (1.18–2.37)	0.004
Q_2_-Q_3_	1.07 (0.76–1.51)	0.697
>Q_3_	Ref	
Bicarbonate T0	1.00 (0.97–1.02)	0.737
ΔBicarbonate		
≤ Q_1_	Ref	
Q_1_-Q_2_	1.14 (0.82–1.57)	0.429
Q_2_-Q_3_	1.18 (0.86–1.62)	0.311
>Q_3_	1.00 (0.72–1.39)	0.991
ΔBicarbonate	1.01 (0.98–1.05)	0.450
Age ≥ 70 years (*n* = 2,017)		
Bicarbonate T0		
≤ Q_1_	1.28 (1.01–1.61)	0.043
Q_1_-Q_2_	1.09 (0.84–1.41)	0.510
Q_2_-Q_3_	0.91 (0.72–1.15)	0.423
>Q_3_	Ref	
Bicarbonate T0	0.98 (0.96–0.99)	0.024
ΔBicarbonate		
≤ Q_1_	Ref	
Q_1_-Q_2_	1.56 (1.19–2.04)	0.001
Q_2_-Q_3_	1.58 (1.21–2.06)	< 0.001
>Q_3_	1.65 (1.28–2.14)	< 0.001
ΔBicarbonate	1.05 (1.02–1.08)	< 0.001
CCI < 5 (*n* = 2,009)		
Bicarbonate T0		
≤ Q_1_	1.23 (0.87–1.75)	0.244
Q_1_-Q_2_	1.36 (0.96–1.93)	0.083
Q_2_-Q_3_	0.98 (0.70–1.38)	0.905
>Q_3_	Ref	
Bicarbonate T0	0.99 (0.96–1.02)	0.342
ΔBicarbonate		
≤ Q_1_	Ref	
Q_1_-Q_2_	1.18 (0.85–1.64)	0.315
Q_2_-Q_3_	1.10 (0.80–1.50)	0.567
>Q_3_	1.00 (0.72–1.38)	0.987
ΔBicarbonate	1.01 (0.98–1.04)	0.468
CCI ≥ 5 (*n* = 2,039)		
Bicarbonate T0		
≤ Q_1_	1.22 (0.97–1.54)	0.095
Q_1_-Q_2_	1.14 (0.88–1.48)	0.329
Q_2_-Q_3_	0.95 (0.75–1.19)	0.643
>Q_3_	Ref	
Bicarbonate T0	0.98 (0.97–1.00)	0.074
ΔBicarbonate		
≤ Q_1_	Ref	
Q_1_-Q_2_	1.57 (1.20–2.06)	< 0.001
Q_2_-Q_3_	1.63 (1.25–2.12)	< 0.001
>Q_3_	1.66 (1.29–2.15)	< 0.001
ΔBicarbonate	1.05 (1.02–1.07)	< 0.001
Thrombolysis = No (*n* = 3,506)		
Bicarbonate T0		
≤ Q_1_	1.24 (1.01–1.52)	0.038
Q_1_-Q_2_	1.19 (0.96–1.48)	0.121
Q_2_-Q_3_	0.95 (0.78–1.16)	0.631
>Q_3_	Ref	
Bicarbonate T0	0.98 (0.97–1.00)	0.065
ΔBicarbonate		
≤ Q_1_	Ref	
Q_1_-Q_2_	1.30 (1.05–1.61)	0.016
Q_2_-Q_3_	1.41 (1.14–1.73)	0.001
>Q_3_	1.32 (1.08–1.63)	0.008
ΔBicarbonate	1.03 (1.01–1.05)	0.003
Thrombolysis = Yes (*n* = 542)		
Bicarbonate T0		
≤ Q_1_	0.98 (0.49–1.95)	0.959
Q_1_-Q_2_	1.84 (0.99–3.43)	0.056
Q_2_-Q_3_	0.98 (0.55–1.75)	0.942
>Q_3_	Ref	
Bicarbonate T0	0.98 (0.92–1.04)	0.455
ΔBicarbonate		
≤ Q_1_	Ref	
Q_1_-Q_2_	2.29 (1.06–4.92)	0.034
Q_2_-Q_3_	1.40 (0.65–3.01)	0.384
>Q_3_	2.12 (1.02–4.40)	0.045
ΔBicarbonate	1.06 (0.99–1.13)	0.106
Antiplatelet agents = No (*n* = 1,589)		
Bicarbonate T0		
≤ Q_1_	1.07 (0.81–1.43)	0.626
Q_1_-Q_2_	1.12 (0.82–1.54)	0.469
Q_2_-Q_3_	0.88 (0.66–1.17)	0.371
>Q_3_	Ref	
Bicarbonate T0	1.01 (0.98–1.03)	0.624
ΔBicarbonate		
≤ Q_1_	Ref	
Q_1_-Q_2_	1.23 (0.90–1.68)	0.187
Q_2_-Q_3_	1.37 (1.02–1.84)	0.036
>Q_3_	1.38 (1.03–1.85)	0.029
ΔBicarbonate	1.04 (1.01–1.07)	0.013
Antiplatelet agents = Yes (*n* = 2,459)		
Bicarbonate T0		
≤ Q_1_	1.36 (1.04–1.77)	0.024
Q_1_-Q_2_	1.37 (1.04–1.80)	0.025
Q_2_-Q_3_	1.02 (0.79–1.32)	0.890
>Q_3_	Ref	
Bicarbonate T0	0.96 (0.94–0.99)	0.001
ΔBicarbonate		
≤ Q_1_	Ref	
Q_1_-Q_2_	1.49 (1.13–1.97)	0.005
Q_2_-Q_3_	1.40 (1.06–1.85)	0.017
>Q_3_	1.31 (1.00–1.73)	0.053
ΔBicarbonate	1.03 (1.00–1.06)	0.062
Anticoagulation agents = No (*n* = 3,254)		
Bicarbonate T0		
≤ Q_1_	1.15 (0.94–1.40)	0.181
Q_1_-Q_2_	1.27 (1.02–1.57)	0.033
Q_2_-Q_3_	0.95 (0.78–1.16)	0.597
>Q_3_	Ref	
Bicarbonate T0	0.99 (0.97–1.01)	0.202
ΔBicarbonate		
≤ Q_1_	Ref	
Q_1_-Q_2_	1.31 (1.06–1.64)	0.014
Q_2_-Q_3_	1.47 (1.19–1.82)	< 0.001
>Q_3_	1.36 (1.11–1.68)	0.004
ΔBicarbonate	1.04 (1.01–1.06)	< 0.001
Anticoagulation agents = Yes (*n* = 794)		
Bicarbonate T0		
≤ Q_1_	2.35 (1.24–4.45)	0.009
Q_1_-Q_2_	1.26 (0.64–2.47)	0.500
Q_2_-Q_3_	1.01 (0.52–1.94)	0.977
>Q_3_	Ref	
Bicarbonate T0	0.93 (0.88–0.98)	0.008
ΔBicarbonate		
≤ Q_1_	Ref	
Q_1_-Q_2_	1.91 (1.01–3.64)	0.049
Q_2_-Q_3_	0.78 (0.37–1.67)	0.528
>Q_3_	1.40 (0.74–2.66)	0.301
ΔBicarbonate	1.01 (0.95–1.08)	0.683

Ref, Reference; HR, Hazard Ratio; CI, Confidence Interval; CCI, Charlson comorbidity index.

In association between bicarbonate T0 and 30–day mortality adjusted for age, gender, ethnicity, ventilation, vasopressor, hyperlipidemia, atrial fibrillation, heart rate, hemoglobin, RDW, CCI, SAPSII, CABG, thrombolysis, antiplatelet agents, and anticoagulation agents if not stratified.

In association between ΔBicarbonate and 30-day mortality adjusted for age, gender, ethnicity, ventilation, vasopressor, hyperlipidemia, atrial fibrillation, diastolic blood pressure, RDW, glucose, CCI, SAPSII, CABG, thrombolysis, antiplatelet agents, and anticoagulation agents if not stratified.

Among participants who did not receive thrombolysis, those with bicarbonate levels at or below the first quartile T0 ≤ Q_1_ had a 24% higher risk of 30-day mortality (HR = 1.24, 95%CI: 1.01–1.52). Similarly, those with changes in bicarbonate levels between the first and second quartiles (Δbicarbonate of Q1–Q2), second and third quartiles (Δbicarbonate of Q2–Q3), or above the third quartile (Δbicarbonate >Q3) had elevated risks of 30-day mortality, with hazard ratios (HRs) of 1.30 (95% CI: 1.05–1.61), 1.41 (95%CI: 1.14–1.73), and 1.32 (95%CI: 1.08–1.63), respectively. An increased risk of 30-day mortality in patients with acute ischemic stroke was elevated with the increase of Δbicarbonate in participants who did not receive thrombolysis (HR = 1.03, 95%CI: 1.01–1.05). Among patients who received thrombolysis, those with Δbicarbonate of Q_1_-Q_2_ (HR = 2.29, 95%CI: 1.06–4.92) or Δbicarbonate >Q_3_ (HR = 2.12, 95%CI: 1.02–4.40) had an increased risk of 30-day mortality in patients with acute ischemic stroke. Among people who did not receive antiplatelet agents, Δbicarbonate of Q_2_-Q_3_ (HR = 1.37, 95%CI: 1.02–1.84) or Δbicarbonate >Q_3_ (HR = 1.38, 95%CI: 1.03–1.85) were associated with an increased risk of 30-day mortality in patients with acute ischemic stroke; and patients with higher Δbicarbonate were correlated with an increased risk of 30-day mortality (HR = 1.04, 95%CI: 1.01–1.07). In people who received antiplatelet agents, bicarbonate T0 ≤ Q_1_ (HR = 1.36, 95%CI: 1.04–1.77), bicarbonate T0 of Q_2_-Q_3_ (HR = 1.37, 95%CI: 1.04–1.80), Δbicarbonate of Q_1_-Q_2_ (HR = 1.49, 95%CI: 1.13–1.97), and Δbicarbonate of Q_2_-Q_3_ (HR = 1.40, 95%CI: 1.06–1.85) were correlated with an increased risk of 30-day mortality in patients with acute ischemic stroke. We also observed that bicarbonate T0 of Q_2_-Q_3_ (HR = 1.27, 95%CI: 1.042–1.57), Δbicarbonate of Q_1_-Q_2_ (HR = 1.31, 95%CI: 1.06–1.64), Δbicarbonate of Q_2_-Q_3_ (HR = 1.47, 95%CI: 1.19–1.82), or Δbicarbonate >Q_3_ (HR = 1.36, 95%CI: 1.11–1.68) were linked with an elevated risk of 30-day mortality in acute ischemic stroke patients who did not receive anticoagulation agents. An elevated level of Δbicarbonate was linked with an increased risk of 30-day mortality in acute ischemic stroke patients who did not receive anticoagulation agents (HR = 1.04, 95%CI: 1.01–1.06). Bicarbonate T0 ≤ Q_1_ (HR = 2.35, 95%CI: 1.24–4.45) and Δbicarbonate of Q_1_-Q_2_ (HR = 1.91, 95%CI: 1.01–3.64) were linked with an elevated risk of 30-day mortality in acute ischemic stroke patients receiving anticoagulation agents. A decreased risk of 30-day mortality in acute ischemic stroke patients who received anticoagulation agents was found in those with higher bicarbonate T0 (HR = 0.93, 95%CI: 0.88–0.98) (Table 5).

## Discussion

The current study assessed the relationship between the bicarbonate levels measured from 0 to 24 h after admission to the ICU and the bicarbonate level changes with 30-day mortality in patients with acute ischemic stroke. The data revealed that bicarbonate T0 ≤ Q_1_ or bicarbonate T0 of Q_2_-Q_3_ were associated with an increased risk of 30-day mortality in patients with acute ischemic stroke. Δbicarbonate of Q_1_-Q_2_, Δbicarbonate of Q_2_-Q_3_, and Δbicarbonate >Q_3_ were correlated with an elevated risk of 30-day mortality in acute ischemic stroke patients. The findings of our study might provide a reference for the management of the prognosis of acute ischemic stroke patients in the ICU.

Bicarbonate is an essential marker that plays an important role in regulating body fluids, acid-base balance, and participation in life activities (19). A low bicarbonate concentration usually represents metabolic acidosis, and low bicarbonate levels may cause astrocyte dysfunction, which was negatively associated with the outcome of stroke patients (20). Another study indicated that lower baseline bicarbonate concentrations were associated with a higher mortality risk among critically ill patients with ischemic cardiogenic shock (21). Lower serum bicarbonate concentrations were found to be significantly associated with higher cardiovascular disease mortality in type 2 diabetes (22). Evidence showed that a low serum bicarbonate level was an independent risk factor for kidney disease progression and mortality in heart failure patients (23). Our study found low baseline bicarbonate levels correlated with an elevated risk of 30-day mortality in patients with acute ischemic stroke. Low serum bicarbonate levels indicated metabolic acidosis, which is a kind of disorder associated with increased mortality, as it is implicated in multiple complications, including cardiac dysfunction, hypotension, and an increased risk of infection (24–26). Bicarbonate is involved in endothelial function, which is one of the pathological mechanisms involved in the development of ischemic stroke (6, 7, 27). It was produced from carbonic anhydrases, which regulated the Neurovascular Unit cells *in vitro* and *in vivo* models of stroke pathology (28). The patients with increased Δbicarbonate had a poor prognosis. The possible reason might be that increased Δbicarbonate indicated the bicarbonate concentration showed a decreasing trend during ICU admission, which usually reflects underlying metabolic acidosis (14), and this might lead to the severity of acute ischemic stroke. Acidosis modulates a wide range of inflammatory gene expression in endothelial cells and regulates endothelial cell adhesion (29), which, in turn, contributes to leukocyte infiltration and plasma leakage with subsequent tissue damage.

In addition, the change in bicarbonate levels was associated with an increased risk of 30-day mortality in acute ischemic stroke patients. These results underscore the importance of monitoring not only the baseline bicarbonate levels but also changes in bicarbonate levels over time. Clinicians should provide special interventions for patients with a decreased trend in the bicarbonate levels. The subgroup analysis revealed that, for patients under 70 years of age, attention should be given to the baseline bicarbonate level, while for patients 70 years of age and older, both the baseline bicarbonate level and changes in bicarbonate levels should be monitored. The Charlson comorbidity index (CCI) is a validated and straightforward method for evaluating the risk of death from comorbid diseases. It has been widely used to predict the prognosis and survival of patients with different diseases (30). In our study, the association between changes in the bicarbonate level and 30-day mortality in acute ischemic stroke patients was identified in those with CCI ≥ 5, suggesting that patients with more comorbid diseases should pay attention to the change in the bicarbonate levels. For patients who received thrombolysis or did not receive antiplatelet agent treatments, dynamic bicarbonate levels should be detected, and those with decreased bicarbonate levels require special care and treatments.

The present study involved a large sample size and found an association between the change in bicarbonate level and the prognosis of patients with acute ischemic stroke. The findings of our study have potential implications for the clinical management of acute ischemic stroke patients. Our findings highlight that the association between bicarbonate levels and the prognosis of patients with acute ischemic stroke may influence clinical decision-making regarding the dose and level of acidosis correction. Sequential monitoring of bicarbonate concentrations may be useful in predicting the prognosis of patients with acute ischemic stroke. In addition, low bicarbonate levels might be associated with a poor prognosis for patients with acute ischemic stroke, which reminds clinicians to be careful with other modifiable factors associated with the prognosis of patients with acute ischemic stroke and provide timely interventions for these patients.

There were several limitations to this study. First, the data were collected from a single-center database, which may limit the generalizability of the results to other populations. Therefore, caution should be exercised when interpreting and applying our findings to other settings. Second, the data on liquid input during the ICU stay, the location and size of the infarction, and stroke etiology were missing or not reported, which might influence the results. Third, the last known well time and the delayed presentation of patients with medical attention were not reported. Fourth, the deep mechanisms underlying the results were not explored. Moreover, the data of the study population were collected from the MIMIC III and MIMIC IV databases and consisted of patients with acute ischemic stroke who were admitted to the ICU. Therefore, caution should be exercised when generalizing our findings to the general population of patients with acute ischemic stroke, as the characteristics of ICUS patients may differ from those of the general population of patients. Finally, blood samples were not obtained at the same time point for all patients, and the difference in the time interval between T0 and T1 may have varied between the patients, which might affect the results. Therefore, further studies are needed to confirm the findings of our study and to determine the optimal timing of bicarbonate level measurements in patients with acute ischemic stroke.

## Conclusions

Our study assessed the relationship between the serum bicarbonate levels and their changes with 30-day mortality in patients with acute ischemic stroke. The results indicated that low baseline bicarbonate levels and decreased bicarbonate levels during ICU stay were associated with a high risk of 30-day mortality in acute ischemic stroke. The findings highlighted the importance of detecting bicarbonate levels and monitoring changes in acute ischemic stroke patients. Special interventions should be provided for those with low baseline bicarbonate levels or/and decreased bicarbonate levels.

## Data availability statement

Publicly available datasets were analyzed in this study. This data can be found here: MIMIC-III: https://www.physionet.org/content/mimiciii/1.4/ and MIMIC-IV: https://www.physionet.org/content/mimiciv/2.2/.

## Ethics statement

The studies involving human participants were reviewed and approved by Beth Israel Deaconess Medical Center (Boston, MA) and the Massachusetts Institute of Technology (Cambridge, MA). This study did not require individual patient consent due to the anonymization of the health information.

## Author contributions

XH and YZ designed the study, collected, analyzed, and interpreted the data. XH wrote the manuscript. YZ critically reviewed, edited, and approved the manuscript. Both authors read and approved the final version of the manuscript.
