# Supplementary material for: Relationship between serum bicarbonate levels and the risk of death within 30 days in ICU patients with acute ischemic stroke
Source: Front Neurol. 2023 May 24;14:1125359. doi: 10.3389/fneur.2023.1125359 (PMC10246426; doi:10.3389/fneur.2023.1125359)
Supplement: Supplementary file 1 [file Table_1.DOCX]

Supplementary Table 1 The missing values of variables analyzed

| Variables | Missing values (n) | Missing values (%) |
| --- | --- | --- |
| Creatinine | 2 | 0.05% |
| BUN | 2 | 0.05% |
| Glucose | 4 | 0.10% |
| Systolic | 34 | 0.84% |
| WBC | 35 | 0.86% |
| Platelet | 36 | 0.89% |
| Hemoglobin | 40 | 0.99% |
| RDW | 46 | 1.14% |
| Diastolic | 47 | 1.16% |
| Heart rate | 55 | 1.36% |
| SPO_2_ | 77 | 1.90% |
| Temperature | 136 | 3.36% |
| Respiratory rate | 158 | 3.90% |

BUN: blood urea nitrogen, WBC: white blood cell, RDW: red cell distribution width, SPO_2_: oxygen saturation

Supplementary Table 2 Sensitivity analysis comparing the data before and after missing value interpolation

| Variables | Total (n=8096) | After interpolation (n=4048) | Before interpolation (n=4048) | Statistics | *P* |
| --- | --- | --- | --- | --- | --- |
| Age, years, Mean ± SD | 67.93 ± 15.57 | 67.93 ± 15.57 | 67.93 ± 15.57 | t=0.00 | 1.000 |
| Gender, n (%) |  |  |  | χ^2^=0.000 | 1.000 |
| Female | 4068 (50.25) | 2034 (50.25) | 2034 (50.25) |  |  |
| Male | 4028 (49.75) | 2014 (49.75) | 2014 (49.75) |  |  |
| Ethnicity, n (%) |  |  |  | χ^2^=0.000 | 1.000 |
| Black | 846 (10.45) | 423 (10.45) | 423 (10.45) |  |  |
| Others | 960 (11.86) | 480 (11.86) | 480 (11.86) |  |  |
| Unknown | 992 (12.25) | 496 (12.25) | 496 (12.25) |  |  |
| White | 5298 (65.44) | 2649 (65.44) | 2649 (65.44) |  |  |
| Ventilation, n (%) |  |  |  | χ^2^=0.000 | 1.000 |
| No | 2734 (33.77) | 1367 (33.77) | 1367 (33.77) |  |  |
| Yes | 5362 (66.23) | 2681 (66.23) | 2681 (66.23) |  |  |
| Vasopressor, n (%) |  |  |  | χ^2^=0.000 | 1.000 |
| No | 5532 (68.33) | 2766 (68.33) | 2766 (68.33) |  |  |
| Yes | 2564 (31.67) | 1282 (31.67) | 1282 (31.67) |  |  |
| Coronary artery disease, n (%) |  |  |  | χ^2^=0.000 | 1.000 |
| No | 5536 (68.38) | 2768 (68.38) | 2768 (68.38) |  |  |
| Yes | 2560 (31.62) | 1280 (31.62) | 1280 (31.62) |  |  |
| Congestive heart failure, n (%) |  |  |  | χ^2^=0.000 | 1.000 |
| No | 5944 (73.42) | 2972 (73.42) | 2972 (73.42) |  |  |
| Yes | 2152 (26.58) | 1076 (26.58) | 1076 (26.58) |  |  |
| Peripheral vascular disease, n (%) |  |  |  | χ^2^=0.000 | 1.000 |
| No | 6936 (85.67) | 3468 (85.67) | 3468 (85.67) |  |  |
| Yes | 1160 (14.33) | 580 (14.33) | 580 (14.33) |  |  |
| Hyperlipidemia, n (%) |  |  |  | χ^2^=0.000 | 1.000 |
| No | 4400 (54.35) | 2200 (54.35) | 2200 (54.35) |  |  |
| Yes | 3696 (45.65) | 1848 (45.65) | 1848 (45.65) |  |  |
| Hypertension, n (%) |  |  |  | χ^2^=0.000 | 1.000 |
| No | 4564 (56.37) | 2282 (56.37) | 2282 (56.37) |  |  |
| Yes | 3532 (43.63) | 1766 (43.63) | 1766 (43.63) |  |  |
| Chronic kidney disease, n (%) |  |  |  | χ^2^=0.000 | 1.000 |
| No | 6750 (83.37) | 3375 (83.37) | 3375 (83.37) |  |  |
| Yes | 1346 (16.63) | 673 (16.63) | 673 (16.63) |  |  |
| Atrial fibrillation, n (%) |  |  |  | χ^2^=0.000 | 1.000 |
| No | 5420 (66.95) | 2710 (66.95) | 2710 (66.95) |  |  |
| Yes | 2676 (33.05) | 1338 (33.05) | 1338 (33.05) |  |  |
| Malignant cancer, n (%) |  |  |  | χ^2^=0.000 | 1.000 |
| No | 7416 (91.60) | 3708 (91.60) | 3708 (91.60) |  |  |
| Yes | 680 (8.40) | 340 (8.40) | 340 (8.40) |  |  |
| Diabetes, n (%) |  |  |  | χ^2^=0.000 | 1.000 |
| No | 5466 (67.51) | 2733 (67.51) | 2733 (67.51) |  |  |
| Yes | 2630 (32.49) | 1315 (32.49) | 1315 (32.49) |  |  |
| Systolic blood pressure, mmHg, Mean ± SD | 137.36 ± 27.10 | 137.42 ± 27.07 | 137.31 ± 27.13 | t=0.17 | 0.863 |
| Diastolic blood pressure, mmHg, Mean ± SD | 72.33 ± 18.10 | 72.38 ± 18.08 | 72.28 ± 18.13 | t=0.25 | 0.802 |
| Respiratory rate, bpm, Mean ± SD | 18.62 ± 5.03 | 18.63 ± 5.00 | 18.61 ± 5.07 | t=0.13 | 0.897 |
| Heart rate, bpm, Mean ± SD | 83.52 ± 18.10 | 83.49 ± 18.06 | 83.56 ± 18.15 | t=-0.19 | 0.852 |
| Temperature, ℃, Mean ± SD | 36.72 ± 0.81 | 36.72 ± 0.81 | 36.72 ± 0.82 | t=0.11 | 0.910 |
| SPO_2_, %, Mean ± SD | 97.63 ± 2.59 | 97.62 ± 2.58 | 97.63 ± 2.60 | t=-0.18 | 0.861 |
| WBC, K/uL, M (Q_1_, Q_3_) | 10.50 (8.00, 13.70) | 10.40 (8.00, 13.70) | 10.50 (8.00, 13.80) | Z=0.202 | 0.840 |
| Platelet, K/uL, M (Q_1_, Q_3_) | 208.00 (158.00, 269.00) | 209.00 (158.00, 268.71) | 208.00 (158.00, 269.00) | Z=-0.118 | 0.906 |
| Hemoglobin, g/dL, Mean ± SD | 11.54 ± 2.26 | 11.54 ± 2.26 | 11.53 ± 2.27 | t=0.13 | 0.900 |
| RDW, %, Mean ± SD | 14.53 ± 1.97 | 14.53 ± 1.96 | 14.54 ± 1.97 | t=-0.07 | 0.945 |
| BUN, mg/dL, M (Q_1_, Q_3_) | 18.00 (13.00, 26.00) | 18.00 (13.00, 26.00) | 18.00 (13.00, 26.00) | Z=0.020 | 0.984 |
| Creatinine, mg/dL, M (Q_1_, Q_3_) | 0.90 (0.70, 1.30) | 0.90 (0.70, 1.26) | 0.90 (0.70, 1.30) | Z=-0.017 | 0.986 |
| Bicarbonate T0, mEq/L, Mean ± SD | 23.24 ± 3.90 | 23.24 ± 3.90 | 23.24 ± 3.90 | t=0.00 | 1.000 |
| Bicarbonate T1, mEq/L. Mean ± SD | 23.62 ± 3.77 | 23.62 ± 3.77 | 23.62 ± 3.77 | t=0.00 | 1.000 |
| Glucose, mg/Dl, M (Q_1_, Q_3_) | 128.00 (105.00, 164.00) | 128.00 (105.00, 164.00) | 128.00 (105.00, 164.00) | Z=-0.013 | 0.989 |
| CCI score, M (Q_1_, Q_3_) | 5.00 (3.00, 7.00) | 5.00 (3.00, 7.00) | 5.00 (3.00, 7.00) | Z=0.000 | 1.000 |
| SAPSII score, M (Q_1_, Q_3_) | 33.00 (26.00, 42.00) | 33.00 (26.00, 42.00) | 33.00 (26.00, 42.00) | Z=0.000 | 1.000 |
| SOFA score, M (Q_1_, Q_3_) | 4.00 (2.00, 6.00) | 4.00 (2.00, 6.00) | 4.00 (2.00, 6.00) | Z=0.000 | 1.000 |
| qSOFA score, M (Q_1_, Q_3_) | 2.00 (1.00, 2.00) | 2.00 (1.00, 2.00) | 2.00 (1.00, 2.00) | Z=0.000 | 1.000 |
| SIRS score, M (Q_1_, Q_3_) | 1.00 (0.00, 1.00) | 1.00 (0.00, 1.00) | 1.00 (0.00, 1.00) | Z=0.000 | 1.000 |
| CABG, n (%) |  |  |  | χ^2^=0.000 | 1.000 |
| No | 7916 (97.78) | 3958 (97.78) | 3958 (97.78) |  |  |
| Yes | 180 (2.22) | 90 (2.22) | 90 (2.22) |  |  |
| Thrombolysis, n (%) |  |  |  | χ^2^=0.000 | 1.000 |
| No | 7012 (86.61) | 3506 (86.61) | 3506 (86.61) |  |  |
| Yes | 1084 (13.39) | 542 (13.39) | 542 (13.39) |  |  |
| Antiplatelet agents, n (%) |  |  |  | χ^2^=0.000 | 1.000 |
| No | 3178 (39.25) | 1589 (39.25) | 1589 (39.25) |  |  |
| Yes | 4918 (60.75) | 2459 (60.75) | 2459 (60.75) |  |  |
| Anticoagulation agents, n (%) |  |  |  | χ^2^=0.000 | 1.000 |
| No | 6508 (80.39) | 3254 (80.39) | 3254 (80.39) |  |  |
| Yes | 1588 (19.61) | 794 (19.61) | 794 (19.61) |  |  |
| LOS, days, M (Q_1_, Q_3_) | 3.57 (1.84, 7.51) | 3.57 (1.84, 7.51) | 3.57 (1.84, 7.51) | Z=0.000 | 1.000 |
| Follow-up time 0, days, Mean ± SD | 25.88 ± 8.54 | 25.88 ± 8.54 | 25.88 ± 8.54 | t=-0.00 | 1.000 |
| Expire flag, n (%) |  |  |  | χ^2^=0.000 | 1.000 |
| Death | 6344 (78.36) | 3172 (78.36) | 3172 (78.36) |  |  |
| Survival | 1752 (21.64) | 876 (21.64) | 876 (21.64) |  |  |

SD: standard deviation, M: Median, Q_1_:1st Quartile, Q_3_: 3st Quartile. CABG: coronary artery bypass grafting, SPO_2_: oxygen saturation, WBC: white blood cell, RDW: red cell distribution width, BUN: blood urea nitrogen, CCI: Charlson comorbidity index, SAPSII: simplified acute physiology score II, SOFA: sequential organ failure assessment, qSOFA: quick sequential organ failure assessment, SIRS: systemic inflammatory response syndrome, LOS: length of stay

# Supplementary Table 3 Association between bicarbonate T0 and 30-day mortality in patients with acute ischemic stroke

| Variables | β | S.E | χ^2^ | HR (95%CI) | *P* |
| --- | --- | --- | --- | --- | --- |
| Bicarbonate T0 | -0.016 | 0.008 | 4.195 | 0.98 (0.97-0.99) | 0.041 |
| Age | 0.023 | 0.003 | 58.138 | 1.02 (1.02-1.03) | <0.001 |
| Gender |  |  |  |  |  |
| Female |  |  |  | Ref |  |
| Male | 0.067 | 0.070 | 0.936 | 1.07 (0.93-1.23) | 0.333 |
| Ethnicity |  |  |  |  |  |
| Black | -0.289 | 0.129 | 4.977 | 0.75 (0.58-0.97) | 0.026 |
| Others | 0.055 | 0.114 | 0.230 | 1.06 (0.84-1.32) | 0.632 |
| Unknown | 0.521 | 0.093 | 31.592 | 1.68 (1.40-2.02) | <0.001 |
| White |  |  |  | Ref |  |
| Ventilation |  |  |  |  |  |
| No |  |  |  | Ref |  |
| Yes | 0.782 | 0.098 | 63.284 | 2.19 (1.80-2.65) | <0.001 |
| Vasopressor |  |  |  |  |  |
| No |  |  |  | Ref |  |
| Yes | 0.242 | 0.079 | 9.440 | 1.27 (1.09-1.49) | 0.002 |
| Hyperlipidemia |  |  |  |  |  |
| No |  |  |  | Ref |  |
| Yes | -0.218 | 0.071 | 9.453 | 0.80 (0.70-0.92) | 0.002 |
| Atrial fibrillation |  |  |  |  |  |
| No |  |  |  | Ref |  |
| Yes | 0.196 | 0.074 | 7.002 | 1.22 (1.05-1.41) | 0.008 |
| Diastolic blood pressure | 0.005 | 0.002 | 6.329 | 1.01 (1.01-1.01) | 0.012 |
| Heart rate | 0.004 | 0.002 | 4.401 | 1.01 (1.01-1.01) | 0.036 |
| RDW | 0.047 | 0.016 | 8.956 | 1.05 (1.02-1.08) | 0.003 |
| CCI score | 0.087 | 0.013 | 43.729 | 1.09 (1.06-1.12) | <0.001 |
| SAPSII score | 0.030 | 0.003 | 101.000 | 1.03 (1.02-1.04) | <0.001 |
| CABG |  |  |  |  |  |
| No |  |  |  | Ref |  |
| Ye | -1.479 | 0.341 | 18.864 | 0.23 (0.12-0.44) | <0.001 |
| Thrombolysis |  |  |  |  |  |
| No |  |  |  | Ref |  |
| Yes | -0.331 | 0.114 | 8.462 | 0.72 (0.57-0.90) | 0.004 |
| Antiplatelet agents |  |  |  |  |  |
| No |  |  |  | Ref |  |
| Yes | -0.186 | 0.070 | 7.019 | 0.83 (0.72-0.95) | 0.008 |
| Anticoagulation agents |  |  |  |  |  |
| No |  |  |  | Ref |  |
| Yes | -1.036 | 0.117 | 78.017 | 0.35 (0.28-0.45) | <0.001 |

Ref: Reference, HR: Hazard Ratio, CI: Confidence Interval, RDW: red cell distribution width, CCI: Charlson comorbidity index, SAPSII: simplified acute physiology score II, CABG: coronary artery bypass grafting

# Supplementary Table 4 Association between Δbicarbonate and 30-day mortality in patients with acute ischemic stroke

| Variables | β | S.E | χ^2^ | HR (95%CI) | *P* |
| --- | --- | --- | --- | --- | --- |
| Δbicarbonate | 0.033 | 0.010 | 10.457 | 1.03 (1.01-1.05) | 0.001 |
| Age | 0.022 | 0.003 | 53.732 | 1.02 (1.02-1.03) | <0.001 |
| Gender |  |  |  |  |  |
| Female |  |  |  | Ref |  |
| Male | 0.060 | 0.070 | 0.737 | 1.06 (0.93-1.22) | 0.391 |
| Ethnicity |  |  |  |  |  |
| Black | -0.282 | 0.129 | 4.761 | 0.75 (0.59-0.97) | 0.029 |
| Others | 0.087 | 0.114 | 0.578 | 1.09 (0.87-1.36) | 0.447 |
| Unknown | 0.527 | 0.092 | 32.528 | 1.69 (1.41-2.03) | <0.001 |
| White |  |  |  | Ref |  |
| Ventilation |  |  |  |  |  |
| No |  |  |  | Ref |  |
| Yes | 0.790 | 0.098 | 64.651 | 2.20 (1.82-2.67) | <0.001 |
| Vasopressor |  |  |  |  |  |
| No |  |  |  | Ref |  |
| Yes | 0.273 | 0.078 | 12.278 | 1.31 (1.13-1.53) | <0.001 |
| Hyperlipidemia |  |  |  |  |  |
| No |  |  |  | Ref |  |
| Yes | -0.207 | 0.071 | 8.451 | 0.81 (0.71-0.93) | 0.004 |
| Atrial fibrillation |  |  |  |  |  |
| No |  |  |  | Ref |  |
| Yes | 0.195 | 0.074 | 6.894 | 1.22 (1.05-1.41) | 0.009 |
| Diastolic blood pressure | 0.004 | 0.002 | 5.783 | 1.01 (1.01-1.01) | 0.016 |
| Heart rate | 0.004 | 0.002 | 4.434 | 1.01 (1.01-1.01) | 0.035 |
| RDW | 0.047 | 0.016 | 8.799 | 1.05 (1.02-1.08) | 0.003 |
| CCI score | 0.085 | 0.013 | 41.658 | 1.09 (1.06-1.12) | <0.001 |
| SAPSII score | 0.031 | 0.003 | 117.935 | 1.03 (1.03-1.04) | <0.001 |
| CABG |  |  |  |  |  |
| No |  |  |  | Ref |  |
| Ye | -1.491 | 0.340 | 19.182 | 0.23 (0.12-0.44) | <0.001 |
| Thrombolysis |  |  |  |  |  |
| No |  |  |  | Ref |  |
| Yes | -0.352 | 0.114 | 9.572 | 0.70 (0.56-0.88) | 0.002 |
| Antiplatelet agents |  |  |  |  |  |
| No |  |  |  | Ref |  |
| Yes | -0.203 | 0.070 | 8.424 | 0.82 (0.71-0.94) | 0.004 |
| Anticoagulation agents |  |  |  |  |  |
| No |  |  |  | Ref |  |
| Yes | -1.044 | 0.117 | 79.328 | 0.35 (0.28-0.44) | <0.001 |

Ref: Reference, HR: Hazard Ratio, CI: Confidence Interval, RDW: red cell distribution width, CCI: Charlson comorbidity index, SAPSII: simplified acute physiology score II, CABG: coronary artery bypass grafting
